# Africa’s Critical Role in Shaping and Implementing the Pandemic Agreement’s PABS Annex in an Era of Fragmentation

**DOI:** 10.1186/s12992-026-01190-3

**Published:** 2026-03-14

**Authors:** Nelson Aghogho Evaborhene

**Affiliations:** Prediagnosis Sierra Leone, Freetown, Sierra Leone

## Abstract

The adoption of the World Health Organization Pandemic Agreement in May 2025 marked a political commitment to stronger global pandemic preparedness. Its credibility, however, depends on the successful negotiation and implementation of the Pathogen Access and Benefit Sharing annex under Article 12, to be finalized by April 2026. The COVID 19 pandemic exposed the failures of voluntary global health mechanisms, as African countries rapidly shared pathogen samples and sequence data yet faced severe delays in access to vaccines, diagnostics, and therapeutics. The PABS annex therefore represents the core test of whether the Agreement can move beyond symbolic equity toward enforceable obligations. This commentary argues that African countries enter the PABS negotiations with an unusual convergence of leverage: early scientific contributions, expanding regulatory and manufacturing capacity, and coordinated diplomacy through the African Union and Africa CDC. In a fragmented multilateral environment dominated by high income state interests, the central challenge is not recognition but conversion of capacity into binding rules. African negotiators have responded by pushing for standardized contracts, traceability of pathogen materials and sequence information, and compliance mechanisms that condition access on enforceable benefit sharing. Drawing on Africa’s institutional readiness, the paper contends that the continent is positioned not merely to influence the PABS annex, but to co-design its operational architecture. Whether this potential is realized will determine if the Pandemic Agreement corrects past inequities or reproduces them under a new legal form.

## Clinical trial number

Not applicable.

## Introduction

In May 2025, Member States of the World Health Organization (WHO) adopted the Pandemic Agreement at the World Health Assembly, concluding nearly three years of complex negotiations. Negotiated under Article 19 of the WHO Constitution, the Agreement represents a landmark commitment to strengthening pandemic preparedness and response globally [1]. As noted by Dr. Tedros Adhanom Ghebreyesus, Director-General of the WHO, its adoption is not only a triumph for public health and science, but also for multilateralism [1]. At a time when rising nationalism and shifting geopolitical dynamics threaten collective action [2], the Agreement signals renewed global resolve to address pandemic threats through cooperation and shared responsibility.

However, the adoption of the Pandemic Agreement is only one phase of the process. The treaty’s legitimacy and effectiveness hinge on the successful negotiation and adoption of its Pathogen Access and Benefit-Sharing (PABS) annex by April 2026 under Article 12 [3]. This article reaffirms states’ sovereign rights over their biological resources while emphasizing the critical need to make pathogen samples and sequence information accessible for research and development. The COVID-19 pandemic laid bare deep inequities in access to medical countermeasures: while some regions secured early access to vaccines and diagnostics, countries in Africa faced long delays and devastating consequences. The PABS annex is thus represent the linchpin of the treaty’s legitimacy and effectiveness.

On 9 July 2025, WHO Member States convened the first meeting of the Intergovernmental Working Group (IGWG) mandated to negotiate the annex. While procedural in form, the meeting marked a turning point. For African countries, the stakes could not be higher. The annex is the litmus test, whether the world can move beyond extractive norms toward a new architecture grounded in equity, sovereignty, and enforceable commitments.

As negotiations move into substantive phases, this commentary argues that African countries enter the process with a rare convergence of moral authority, scientific capability, and institutional readiness, albeit within a fragmented multilateral environment. The continent’s collective diplomatic stance—expressed in the African Union’s Common African Position and shaped through the April 2024 High-Level Ministerial Consultation in Addis Ababa—places it in a strong position not just to influence, but to lead [4].

Yet the continent must navigate a multilateral order historically dominated by high-income states, where domestic and commercial interests often conflict with the objectives of global equity. The challenge therefore is not asserting relevance, but converting demonstrated capacity into binding rules, operational mechanisms, and enforceable guarantees. This moment demands more than inclusion: African leadership, institutions, and infrastructure must be embedded in the rules of a new enforceable global health order. Achieving this requires strategic engagement across three interlinked domains: negotiation, structural integration, and operational implementation.

## From Voluntary Ideals to Binding Obligations

The COVID-19 pandemic laid bare the structural weaknesses and inequities embedded in voluntary global health frameworks. For instance, early in the crisis, African scientific institutions—such as South Africa’s Centre for Epidemic Response and Innovation and Nigeria’s African Centre of Excellence for Genomics of Infectious Diseases —were among the first globally to sequence and publicly share SARS-CoV-2 genomes, playing a critical role in global surveillance [5]. Yet despite these contributions, African countries faced prolonged delays in accessing vaccines and therapeutics. By mid-2021, less than 2% of the continent’s population had received a COVID-19 vaccine dose, despite over 5.7 billion doses administered globally [6].

These disparities reflected a global health system heavily reliant on goodwill and voluntary mechanisms. COVAX, the COVID-19 Vaccines Global Access Facility, was designed to pool resources from wealthy countries to procure and equitably distribute vaccines to low- and middle-income countries. While commendable in ambition, it struggled under the weight of export bans, commercial self-interest, and limited manufacturing diversity, ultimately leaving many low-income regions behind. The experience highlighted those voluntary mechanisms, even when multilateral and well-intentioned, could not reliably safeguard equitable access without binding commitments or structural reforms [7].

Against this backdrop, the PABS annex must mark a deliberate shift from these non-binding approaches to a regime of enforceable commitments. Article 12 of the Pandemic Agreement signals this shift [3], requiring legal contracts with manufacturers to guarantee access to at least 20% of relevant pandemic-related products—half to be donated, and the remainder to be provided at affordable prices. Distribution of these supplies would be coordinated by WHO, with allocation guided by public health risk and explicit safeguards for low- and middle-income countries.

Still, doubts remain as to whether these measures will be sufficient [8]. Scholars of international law have warned that without stronger mechanisms to prevent nationalist hoarding, the PABS framework may replicate the shortcomings of earlier models like COVAX and the Pandemic Influenza Preparedness Framework—delaying the timely provision of countermeasures to WHO until high-income countries have secured their own needs [9].

In response to these risks in April 2024, African ministers emphasized that legal certainty is a prerequisite [4]. For countries to engage equitably and for industry to participate with confidence therefore, the annex must establish clear, enforceable rules for both pathogen contributors and recipients. It must also incorporate binding commitments on technology transfer, regional manufacturing, and benefit-sharing—underpinned by transparent monitoring and accountability mechanisms.

This momentum was reinforced in May 2025 at the adoption of the Pandemic Agreement. African delegations, signalled a clear commitment to equity and enforceable governance in the PABS annex. Speaking for the continent, South Africa highlighted that African countries had long shared pathogens and sequence data without receiving timely or adequate countermeasures, stressing that operational readiness and legally binding safeguards were essential to ensure manufacturer commitments translated into real access during health emergencies [10]. Kenya, Uganda, and Namibia reinforced the need for PABS to allocate resources efficiently and equitably, guided by principles of solidarity and cooperation. These statements underscored the continent’s insistence that the PABS framework must rectify historical inequities while balancing the dual mandate of rapid data sharing and fair benefit distribution.

## Negotiating Sovereignty and Sharing: African Delegations in Action

Following the adoption of the Pandemic Agreement in May 2025, African negotiators have remained actively engaged in the IGWG process to shape the PABS annex at an operational level. In these negotiations, the Africa Group refers to the coalition of WHO African Region Member States negotiating as a coordinated regional bloc, with positions shaped through African Union processes and articulated collectively within WHO negotiating fora. Acting through common submissions and joint interventions, the Africa Group has served as the primary vehicle through which African countries advance shared continental priorities on equity, sovereignty, and enforceable benefit sharing.

In its initial proposals submitted in August 2025 [11], the Africa Group emphasized that the PABS framework must safeguard sovereign rights while aligning with existing international instruments, including the Pandemic Influenza Preparedness Framework, the Convention on Biological Diversity, and the Nagoya Protocol. The proposal stressed the need for clear definitions, operationally enforceable obligations, and mechanisms for equitable access.

These positions were sharpened and publicly reinforced during the fourth session of the IGWG in Geneva in December 2025. Speaking on behalf of the Africa Group and the Group for Equity, Zimbabwe articulated a unified Global South position. The coalition argued that the PABS annex must articulate the full operational details of the system and that negotiations on standardized PABS contracts must begin immediately within the IGWG process rather than being deferred to the Conference of the Parties after ratification [12].

The coalition presented draft standardized contracts covering material transfer within the WHO collaborating laboratory network, transfers outside that network, and access to PABS related sequence databases. Zimbabwe emphasized that these contracts are not administrative formalities but the legal backbone of the system, defining not only benefit sharing obligations but also the rights and responsibilities of providers and users, including terms of access, use, traceability, and accountability. While participation in the PABS system would remain voluntary, access to pathogen materials and sequence information would be conditional on acceptance of contractual terms, a structure explicitly framed as necessary to respect sovereign rights, prevent free riding, and rebuild trust.

African delegations repeatedly grounded their demands in lived experience. Uganda, aligning with the coalition, warned that predictable and equitable benefit sharing is a matter of life or death, citing past outbreaks of Ebola, Marburg, and mpox where access to countermeasures lagged despite early pathogen sharing. Zambia argued that without embedding the full operational details of Article 12 into contracts negotiated before entry into force, legal certainty would be undermined, and the system would remain vulnerable to selective implementation.

Across these interventions, a consistent theme emerged. African negotiators are not resisting sharing obligations. They are resisting a system in which sharing is automatic while benefits remain discretionary, delayed, or politically mediated. The insistence on standardized contracts reflects a strategic effort to hard wire equity into the legal architecture of the Pandemic Agreement rather than leaving it to future negotiations or goodwill.

Negotiations thus reflect the inherent tension between sovereignty and sharing obligations under Article 12. African delegations have consistently pushed back against proposals that weaken equity provisions or create vague legal commitments. Key sticking points include allocation rules, intellectual property rights, regulatory harmonization, and dispute resolution. Despite these challenges, African delegations are actively shaping both the language and practical implementation of a system intended to govern pathogen access and equitable benefit-sharing, establishing a precedent for enforceable and accountable global health governance.

## Designing for Compliance, Not Just Consensus

Equity in the PABS system cannot rest on political goodwill alone. It must be embedded in the system’s architecture through enforceable and transparent mechanisms that hold all stakeholders, including states and private actors, accountable. The African Union has repeatedly underscored the need for safeguards to prevent the exploitation of pathogen materials and digital sequence information [4]. Experience shows how industry have generated billion-dollar products from publicly shared viral sequences while source countries received delayed or negligible benefits. These extractive dynamics have persisted despite initiatives such as the WHO mRNA Technology Transfer Hub in South Africa, where the continued refusal of key patent holders to share technology continue to constrain regional manufacturing capacity [13].

While Article 12.3 of the Pandemic Agreement mandates the development of traceability measures alongside open access to data, it deliberately leaves their design to the IGWG [3]. Traceability of pathogen samples and digital sequence information is widely recognized as essential to credible benefit sharing, yet it poses substantial technical, financial, and logistical challenges. Unlike physical samples, for which the Influenza Virus Traceability Mechanism offers a partial precedent, there is no established global model for tracing the downstream use of genomic data once it enters open repositories. Any workable approach must therefore balance transparency and enforceability with the imperative of rapid data sharing for public health response.

African negotiators have treated this challenge as foundational. In its August 2025 submission to the Intergovernmental Working Group, the Africa Group explicitly identified ethical use, integrity, and traceability of PABS materials and sequence information as core normative principles of the annex. Traceability was positioned alongside sovereign rights, equitable benefit sharing, and mutual accountability, signaling a clear rejection of systems that rely on voluntary disclosure or ex post political pressure. The Africa Group further proposed that access to PABS materials and sequence information be governed by legally binding contracts with the WHO, applicable to all recipients, and supported by a WHO administered tracing and tracking mechanism. Metadata and identifiers linking sequence information to its country of origin and to the PABS system were explicitly required to remain intact, ensuring that scientific utility is preserved while downstream use remains auditable.

At the same time, traceability must be operationally realistic. As Switzer and colleagues caution, enforceability that ignores implementation capacity risks collapse [14]. One option warranting serious consideration is a global digital ledger to record pathogen sharing, access to sequence information, and benefit sharing obligations. Properly designed, such a system could generate an immutable audit trail enabling real time compliance monitoring while limiting selective reporting or data manipulation. Unlike platforms such as GISAID, which enable data sharing without enforcing benefit obligations, a ledger-based system could link access directly to accountability without restricting scientific exchange. Its credibility would depend on decentralization, with nodes operated by WHO, regional bodies, national public health laboratories, and other authorized actors.

Compliance can also be reinforced beyond the PABS system itself. Scientific journals could require disclosure of sequence origins as a condition of publication. Funders could condition grant eligibility on adherence to PABS traceability standards. Governments could mandate disclosure of PABS resource use in patent applications and regulatory submissions. Together, these measures would embed traceability across research, financing, and commercialization pathways, shifting it from an administrative requirement to a governing norm.

Understood this way, traceability is not a constraint on sharing but a prerequisite for trust. By anchoring transparency and accountability in enforceable rules and interoperable systems, African negotiators can advance a PABS model that aligns rapid access with auditable benefit sharing. If operationalized, this approach would move the system beyond consensus language toward compliance driven equity, ensuring that access and benefit sharing function as inseparable obligations rather than parallel promises.

## From Compliance to Capacity: Africa’s Institutional Readiness to Operationalise PABS

Africa’s push for enforceable compliance is grounded in its growing institutional capacity. The COVID 19 pandemic marked a turning point in the continent’s role within global health governance. The Africa Centres for Disease Control and Prevention has emerged as a central coordinating authority, strengthening regional surveillance, logistics, and workforce capacity while facilitating access to medical countermeasures through the Africa Medical Supplies Platform [15]. Under conditions of acute global scarcity, this experience demonstrated that African institutions are capable of designing and operating complex procurement and distribution systems at continental scale.

Building on this foundation, Africa CDC in 2021 launched the Partnership for African Vaccine Manufacturing (Platform for Harmonised African Health Products Manufacturing) with the objective of meeting 60% of the continent’s vaccine demand by 2040 [16]. By mid-2024, 25 vaccine manufacturing initiatives were underway across the continent. Afrigen Biologics in South Africa developed an mRNA vaccine prototype through collaboration with the WHO mRNA Technology Transfer Hub, marking a significant step toward technological autonomy. The Institut Pasteur de Dakar expanded production capacity through long term partnerships supported by CEPI and the European Union. Vacsera in Egypt scaled biomanufacturing infrastructure, while Biovac in South Africa advanced conjugate vaccine production through technology transfer agreements. Together, these initiatives reflect not only industrial ambition but also growing competence in regulatory oversight, quality assurance, and compliance with international standards.

Regulatory convergence has progressed in parallel with manufacturing expansion. The African Medicines Agency is advancing efforts to harmonize regulatory approval processes across the continent, and eight countries have achieved Maturity Level 3 under the WHO Global Benchmarking Tool, indicating functional national regulatory systems aligned with international norms [17]. This progress is critical because regulatory fragmentation has direct consequences for access. During the mpox outbreak in the Democratic Republic of Congo, limited regulatory recognition and fragmented approval pathways delayed access to vaccines by several months, despite urgent epidemiological need. The experience underscored a central lesson: manufacturing capacity without regulatory alignment does not translate into timely access.

In this context, the PABS system has the potential to serve not only as a distribution mechanism but also as a catalyst for regulatory convergence. By linking access to pathogen materials and benefit sharing to defined regulatory benchmarks, PABS can incentivize sustained investment in national regulatory authorities. South Africa’s experience illustrates this dynamic. Engagement with the WHO mRNA Technology Transfer Hub strengthened the capacity of the South African Health Products Regulatory Authority to evaluate novel biologics and mRNA platforms, enhancing both safety oversight and public trust. As additional technologies emerge through PABS, other regulators across the continent will need to develop comparable competencies, reinforcing regulatory coherence at the regional level.

Africa’s readiness is equally evident in pathogen surveillance and data governance. More than 70% of African Union member states now possess domestic sequencing capacity, and over 180,000 SARS CoV 2 genomes have been shared globally [18]. The Africa CDC Pathogen Genomics Initiative, launched in 2020, has enabled rapid detection of multiple outbreaks including mpox. Its successor platform, Africa Genome Archiving for Response and Insight, launched in 2025, aims to provide a continent-wide genomic data system directly linked to public health decision making. These platforms provide the technical substrate for the traceability mechanisms African negotiators are advancing within the PABS framework.

Supply chain coordination has also advanced. The African Union, working with Regional Economic Communities, launched the African Pooled Procurement Mechanism to strengthen coordinated purchasing and logistics [19]. Complementing this architecture is the African Vaccine Manufacturing Accelerator, launched by GAVI in June 2024 in response to the AU’s call for at least 30% of vaccines procured for Africa to be locally produced. The Accelerator provides long term financing and market incentives to support sustainable manufacturing, anchoring Africa’s broader vision of health sovereignty under the New Public Health Order.

## Bridging the Technology Gap: Enforceable Transfers and Negotiating Power

However, without appropriate technology, these initiatives risk falling short. Critics have argued that financial incentives based on outdated market thinking are insufficient [20]. The African Development Bank’s African Pharmaceutical Technology Foundation, launched in 2022 and headquartered in Kigali in 2023, was designed to bridge critical gaps in local manufacturing and innovation. Yet global frameworks, including the adopted Pandemic Agreement, still fall short on enforceable technology-transfer commitments.

While African delegations in Geneva successfully secured recognition of LMIC concerns—pushing for provisions on equitable access, transparency, and regional capacity building—representation alone did not guarantee full negotiating power. Legal, financial, and political asymmetries limited outcomes, evident in the treaty’s final language where once-binding clauses on IP waivers and royalty-free licensing were replaced with vague, non-binding formulations. The Independent Panel on Pandemic Preparedness and Response has warned that such caveats risk inaction, highlighting the gap between diplomatic presence and enforceable outcomes critical for strengthening local manufacturing and health sovereignty [21].

Moreover, the United States’ withdrawal from WHO and its refusal to participate in or adopt the Pandemic Agreement could constrain the treaty’s global reach. These challenges are now intensified in its bilateral arrangements outside multilateral frameworks. Under its America First Global Health Strategy [22], some agreements condition access to health financing on the sharing of pathogen specimens, genetic sequence data, and sensitive health system information. In several cases, such arrangements enable long-term access to national pathogen and surveillance data without enforceable guarantees on benefit sharing or technology transfer. The Kenya–United States agreement illustrates these risks, raising concerns about unclear obligations, reciprocity, and accountability. Such fragmentation weakens negotiating power and erodes the collective safeguards the PABS system seeks to establish [23].

African negotiators face a fundamental tension: pressing for immediate access to medical countermeasures to protect lives in the short term, while securing enforceable technology-transfer commitments to build long-term health sovereignty. Higher-capacity states such as South Africa, Senegal, and Egypt are positioned to advance technology transfer and manufacturing integration, whereas lower-capacity countries including the Democratic Republic of the Congo face infrastructure and regulatory constraints. Without mechanisms to translate collective commitments into coordinated execution, these disparities risk undermining both continental leverage and equitable outcomes.

Building on the African Union’s Common African Position (CAP) on Pandemic Prevention, Preparedness, and Response (February 2024, reinforced August 2025), African negotiators must act decisively, alongside like-minded Global South coalitions, to secure concrete technology-transfer provisions while ensuring timely access to vaccines, therapeutics, and diagnostics. Properly implemented, the PABS annex could simultaneously strengthen Africa’s health security, advance industrial and regulatory capacity, and embed production, trade, and innovation into the continent’s long-term development agenda under AfCFTA and Agenda 2063.

## From Capacity to Authority: Governing Fragmentation and Safeguarding PABS Implementation

Addressing the gaps and tensions highlighted above requires robust governance solutions. To protect continental interests in this environment, African countries should advocate for the formal establishment of a continental PABS Secretariat, jointly led by Africa CDC, the African Medicines Agency, and the African Union Commission, and explicitly mandated within the PABS annex. Legal authority must derive directly from the treaty, enabling the Secretariat to coordinate pathogen access and benefit sharing on behalf of the continent. Formal recognition through African Union legal instruments would embed the Secretariat within continental governance structures, ensuring alignment with regional priorities and operational realities [23].

Calls for a WHO led adoption of the PABS annex in the global south [24] will remain hollow unless anchored in a regional structure led by African institutions. A continental Secretariat does not dilute WHO authority. It strengthens it. WHO remains one of the few institutions capable of enforcing multilateral rules in the face of bilateral pressure, while a unified Africa provides WHO with a coherent and credible regional counterpart. This division of roles enhances legitimacy, consistency, and system resilience rather than creating parallel authority.

Signals of continental resolve are already evident. During the fourth IGWG meeting, Zimbabwe, speaking on behalf of fifty African countries, emphasized that pathogen materials and sequence information should flow through WHO coordinated systems [23]. The Elders have likewise warned that parallel bilateral arrangements risk undermining the foundations of global preparedness currently under construction. These interventions demonstrate a clear willingness to assert leadership and defend multilateral integrity [23].

The Africa Group initial submission to the IGWG provides a strong legal and normative basis for a continental coordinating body. By recognizing the role of relevant regional health governmental institutions and emphasizing the coordinated exercise of sovereign rights through designated national focal points, the submission supports the establishment of a continental PABS Secretariat. Its emphasis on accountability, transparency, and legally binding contracts with WHO reinforces the Secretariat’s potential role in monitoring compliance, enforcing traceability, and coordinating equitable benefit sharing.

Functioning as a regional hub, the Secretariat could integrate Africa’s scientific, regulatory, and manufacturing capacities into the global PABS system. By linking Africa CDC Pathogen Genomics Initiative and RISLNET with global traceability mechanisms, and coordinating with the African Medicines Agency, the African Pharmaceutical Technology Foundation and the Africa Medical Supplies Platform, it would support timely and transparent implementation of treaty obligations. In doing so, it would anchor Africa’s leadership in pathogen governance while strengthening equitable access to vaccines, therapeutics, and diagnostics.

Empowering regional institutions to coordinate benefit sharing, technology transfer, and allocation of countermeasures would enhance both effectiveness and legitimacy. When aligned with the African Continental Free Trade Area, these efforts could support integrated regional value chains, facilitate cross border trade in medical products, and advance broader industrial and development objectives under the African Union Agenda 2063.

## Integrating Africa’s Institutions Into the Global Supply Chain and Logistics Network

To translate pathogen access into the real time delivery of lifesaving tools, African institutions must be embedded within the Pandemic Agreement’s broader implementation architecture, particularly the Global Supply Chain and Logistics (GSCL) Network. Established under Article 13, the GSCL Network is a WHO convened mechanism designed to ensure timely, equitable, and affordable access to pandemic related health products across all phases of preparedness and response. Its effectiveness depends not only on technical coordination, but on sustained regional integration and decision-making authority.

Africa’s capacity to contribute meaningfully to this system is demonstrated by recent operational experience. During the COVID 19 pandemic, the Africa Medical Supplies Platform, launched by the African Union under the leadership of Special Envoy Strive Masiyiwa, centralized continental procurement and facilitated the distribution of diagnostics, therapeutics, and personal protective equipment at scale. Similarly, the African Vaccine Acquisition Trust (AVAT), established by the African Union with support from Africa CDC, Afreximbank, and the United Nations Economic Commission for Africa, demonstrated the continent’s ability to pool demand, mobilize financing, and coordinate cross border vaccine procurement and delivery [25]. Together, these mechanisms showed that African institutions can design and manage complex logistics systems under urgent timelines and severe global supply constraints.

For the GSCL Network to function as intended, it must be interoperable with such regionally owned mechanisms. This requires embedding African representation within its governance structures, ensuring equitable participation across WHO regions, and maintaining transparent decision-making processes. Without this, the Network risks reproducing past patterns where African countries are treated as end users rather than co architects of global supply strategies.

Once established, a continental PABS Secretariat should serve as the primary conduit linking regional logistics, regulatory, and manufacturing systems with the GSCL infrastructure. Working alongside Africa CDC, the Africa Medical Supplies Platform, RISLNET, Regional Economic Communities, and the African Medicines Agency, the Secretariat could align pathogen sharing obligations with regulatory approval pathways, manufacturing readiness, and distribution planning. This linkage is particularly critical during inter pandemic periods, when demand forecasting, stockpile governance, and pre-positioning of supplies determine whether equity can be achieved once a crisis emerges.

At the same time, these achievements should not obscure implementation risks. Delays in finalizing PABS contracts could stall access to countermeasures at the moment of need. Regulatory fragmentation across countries may limit the effective use of shared pathogens and data. Bilateral agreements that bypass multilateral mechanisms could undermine equitable allocation. Capacity heterogeneity across the continent means that lower capacity states may struggle to meet reporting, regulatory, or operational requirements, risking uneven benefit sharing and reinforcing internal disparities.

Addressing these risks will determine whether Africa’s demonstrated institutional capacity translates into a functioning and enforceable PABS system, or whether past failures are repeated under a new legal framework.

## A Shift in the Moral Centre of Global Health

As the world moves toward a new global health order, African institutions cannot remain passive stakeholders. They must act as co-architects of the rules that govern pathogen access and benefit sharing. The continent’s scientific contributions, institutional maturation, and diplomatic coordination provide a credible mandate to shape what follows. Leadership alone, however, is insufficient. In an increasingly fragmented global landscape, leverage matters, and that leverage must be embedded in the legal text, operational mechanisms, and timelines of the PABS annex.

The April 2026 deadline to finalize Article 12 presents a narrow but consequential opportunity to correct structural inequities exposed during COVID 19 before the next pandemic strikes. If pathogens are to be shared globally, then the benefits, technologies, and decision-making authority they generate must also be shared on equitable terms. Africa’s position is grounded in legal demands for certainty, expanding regulatory and manufacturing capacity.

For the Pandemic Agreement to succeed, the rest of the world must respond with binding commitments that make equity a governing principle rather than a political aspiration. Without enforceable contracts, traceable obligations, and predictable allocation rules, PABS risks reproducing the failures of earlier voluntary frameworks.

At the same time, success is not guaranteed. Implementation will hinge on timely finalization contracts, regulatory harmonization across diverse national systems, and alignment of national priorities within and beyond the continent. Sustained political commitment, robust monitoring, and enforceable safeguards will be required to prevent high income countries from dominating access during future emergencies. Failure to address these conditions risks entrenching existing disparities, marginalizing lower capacity states, and undermining the credibility of the Pandemic Agreement itself.

## Data Availability

No datasets were generated or analysed during the current study.
